# Supplementary material for: Biodegradation of Oil by a Newly Isolated Strain Acinetobacter junii WCO-9 and Its Comparative Pan-Genome Analysis
Source: Microorganisms. 2023 Feb 6;11(2):407. doi: 10.3390/microorganisms11020407 (PMC9967506; doi:10.3390/microorganisms11020407)
Supplement: Supplementary file 1 [file microorganisms-11-00407-s001.zip › microorganisms-2167671-supplementary.pdf]

Biodegradation of Oil by a Newly Isolated Strain *Acinetobacter junii* WCO-9 and Its Comparative Pan-Genome Analysis

Shijie Jiang [1,2,*,†], Qingfeng Fan [1,2,†], Zeying Zhang [1], Yunfeng Deng [1,2], Lihong Wang [1], Qilin Dai [1,2], Jin Wang [3], Min Lin [3], Jian Zhou [1,2], Zhijian Long [1,2], Guiqiang He [1,2] and Zhengfu Zhou [3,*]

1 School of Life Science and Engineering, Southwest University of Science and Technology, Mianyang 621010, China
2 Engineering Research Center of Biomass Materials, Ministry of Education, Southwest University of Science and Technology, Mianyang 621010, China
3 Key Laboratory of Agricultural Microbiome (MARA), Biotechnology Research Institute, Chinese Academy of Agricultural Sciences, Beijing 100081, China
* Correspondence: shijiejiang525@163.com (S.J.); zhouzhengfu@caas.cn (Z.Z.)
† These authors contributed equally to this work.

Supplementary Materials

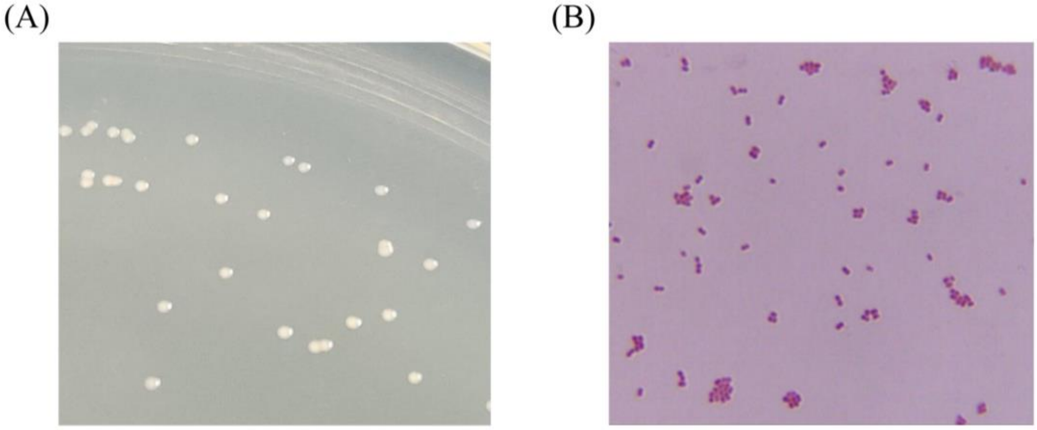

Figure S1. Colony morphology and Gram staining of strain WCO-9

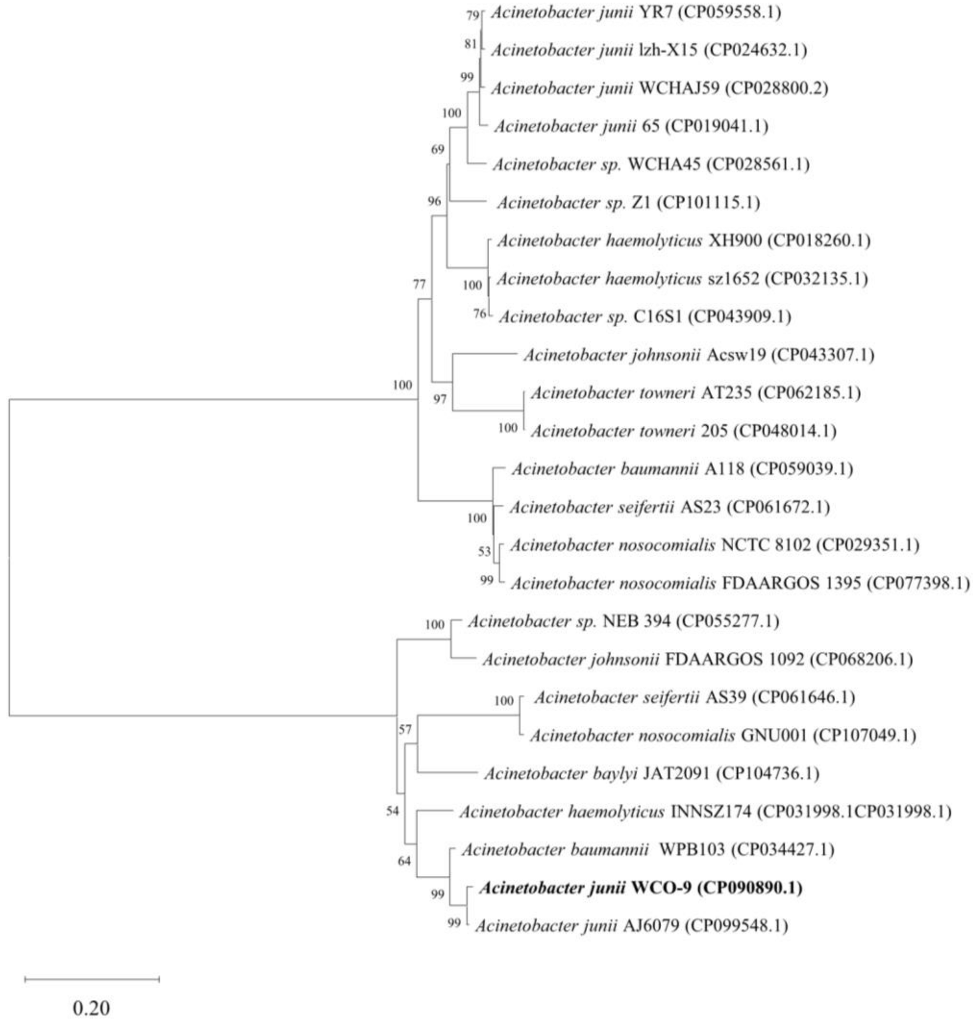

Figure S2. Phylogenetic tree based on the *ileS* gene sequence. This tree was constructed by the neighbor-joining method, and showed the phylogenetic relationship between strain WCO-9 and closely related species.

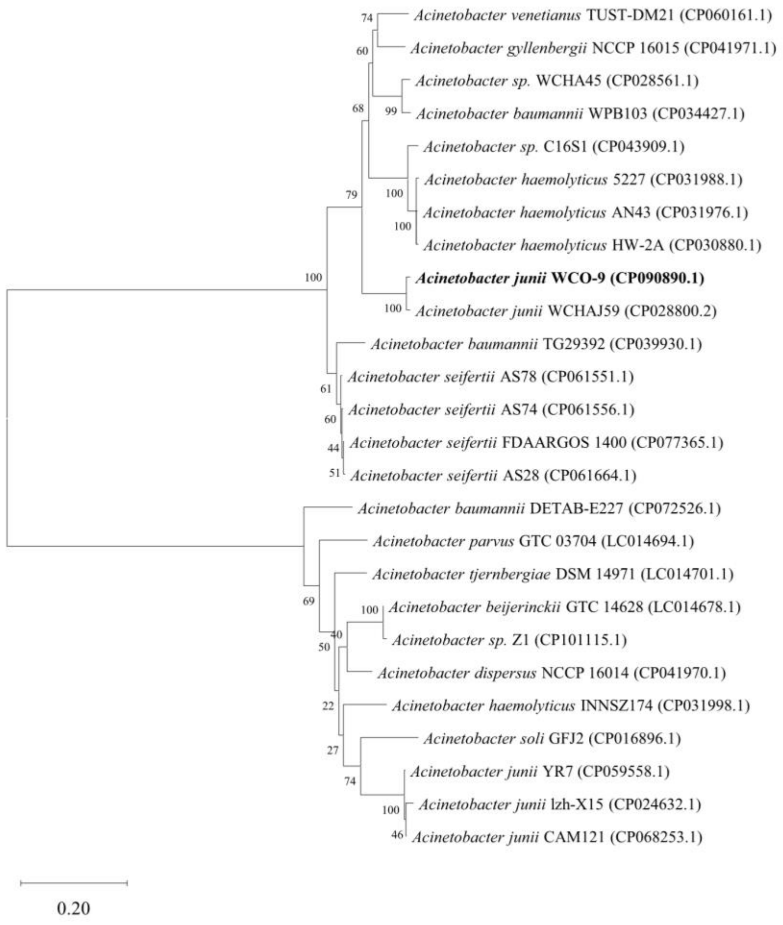

Figure S3. Phylogenetic tree based on the *recA* gene sequence. This tree was constructed by the neighbor-joining method, and showed the phylogenetic relationship between strain WCO-9 and closely related species.

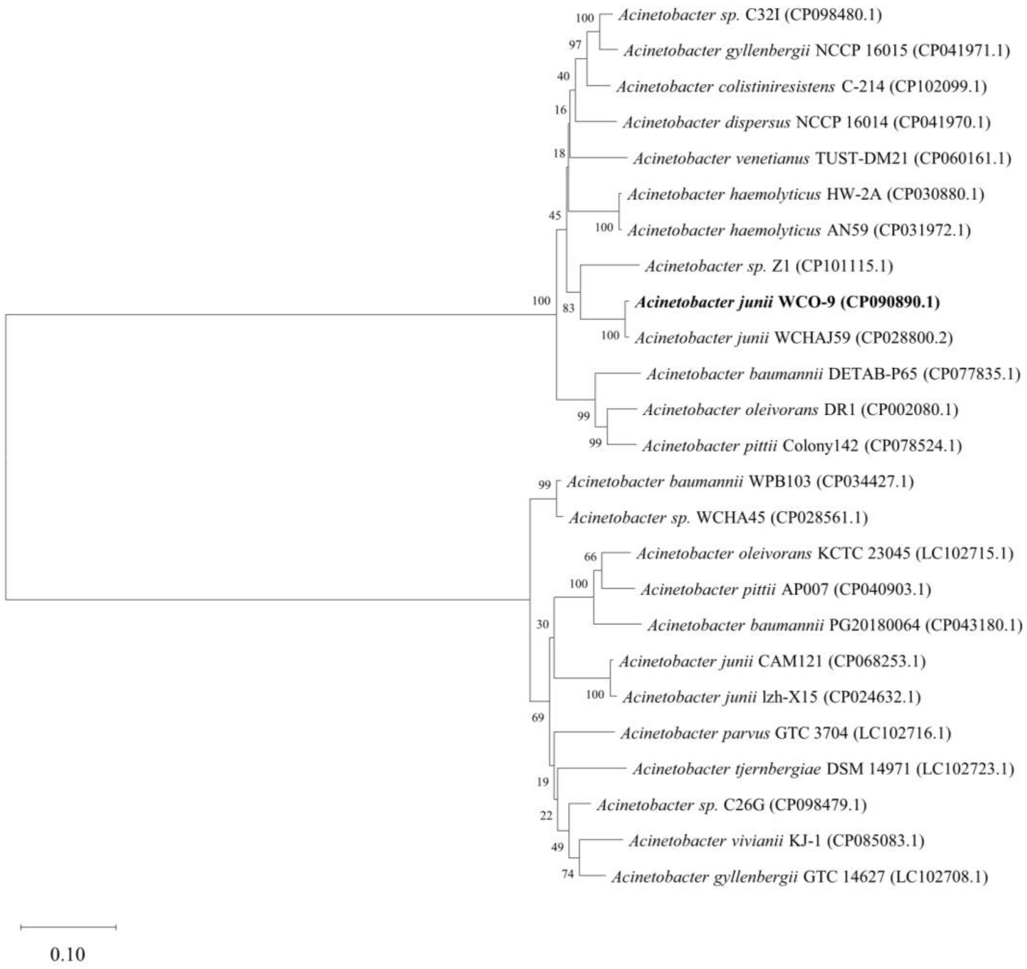

Figure S4. Phylogenetic tree based on the *rpoD* gene sequence. This tree was constructed by the neighbor-joining method, and showed the phylogenetic relationship between strain WCO-9 and closely related species.

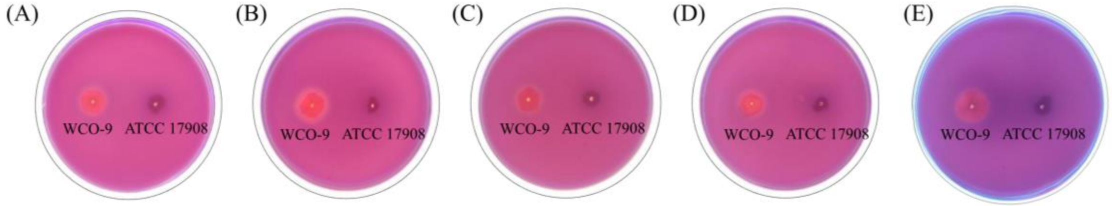

Figure S5. Degradation of five oils by strain WCO-9 and ATCC 17908. (A) Corn oil; (B) Peanut oil; (C) Canola oil; (D) Soybean oil; (E) Olive oil.

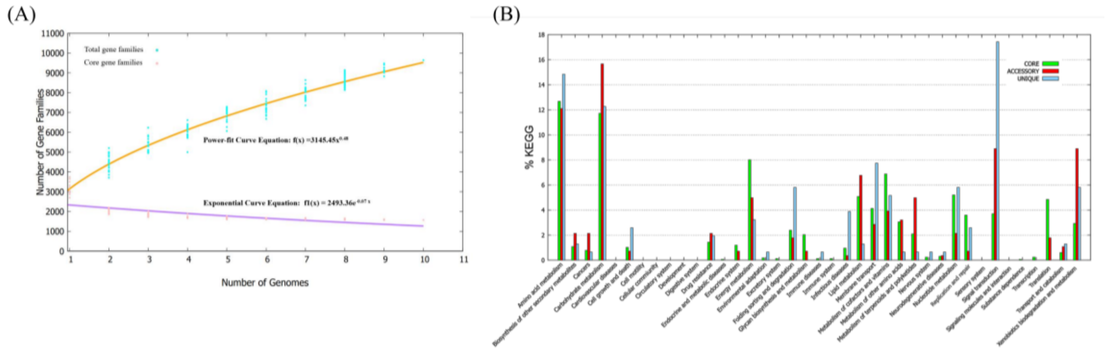

Figure S6. Pan-genome analysis of *A junii* strains. (A) Mathematical modeling of the pan-genome and core genome of *A. junii*. (B) Bar chart showing functional proportions (based on KEGG categories) of different parts of the *A. junii* pan-genome (i.e., core, accessory, unique).
